# Clinical and endoscopic features of checkpoint inhibitor–induced colitis and their association with selective immunosuppressive therapy use

**DOI:** 10.1093/ibd/izag104

**Published:** 2026-08-21

**Authors:** Myrthe R Naber, Mick J M van Eijs, Jildou S Huitema, Jacco J de Haan, Karijn P M Suijkerbuijk, Marijn C Visschedijk, Fiona D M van Schaik

**Affiliations:** Department of Gastroenterology and Hepatology, University Medical Centre Utrecht, Utrecht University, Heidelberglaan 100, Utrecht, 3584 CX, the Netherlands; Department of Oncology, University Medical Centre Utrecht, Utrecht University, Heidelberglaan 100, Utrecht, 3584 CX, the Netherlands; Department of Gastroenterology and Hepatology, University Medical Centre Groningen, Hanzeplein 1, Groningen, 9713 GZ, the Netherlands; Department of Oncology, University Medical Centre Groningen, Hanzeplein 1, Groningen, 9713 GZ, the Netherlands; Department of Oncology, University Medical Centre Utrecht, Utrecht University, Heidelberglaan 100, Utrecht, 3584 CX, the Netherlands; Department of Gastroenterology and Hepatology, University Medical Centre Groningen, Hanzeplein 1, Groningen, 9713 GZ, the Netherlands; Department of Gastroenterology and Hepatology, University Medical Centre Utrecht, Utrecht University, Heidelberglaan 100, Utrecht, 3584 CX, the Netherlands

**Keywords:** checkpoint inhibitor–induced colitis, selective immunosuppressive therapy, endoscopy

## Abstract

**Background and Aims:**

Immune checkpoint–inhibitor (ICI) colitis is a common adverse event that is primarily treated with corticosteroids but may require selective immunosuppressive therapy (SIT). Although endoscopic features have been associated with SIT use, it remains unclear whether these associations differ by ICI type.

**Methods:**

We conducted a 2-center retrospective cohort study and included patients with histologically confirmed ICI colitis. Endoscopic images were reviewed by gastroenterologists specialized in inflammatory bowel disease (IBD) and scored using the Mayo score and the Immune-Mediated Colitis Endoscopic Score (IMCES). Early SIT use was defined as SIT use within 3 weeks after the start of colitis treatment. Features associated with SIT use were stratified by ICI type, and discriminative performance was evaluated using receiver operating characteristic (ROC) analysis.

**Results:**

Among 255 included patients, 38% required SIT. Biochemical parameters associated with early SIT use were C-reactive protein (CRP) (odds ratio (OR), 1.41; P = .005), and albumin (OR, 0.92; P = .020). Endoscopic features associated with SIT use included erythema (OR, 4.09; P = .006) and exudate (OR, 3.24; P = .012), which were limited to patients treated with anti-cytotoxic T-lymphocyte-associated antigen 4–based (aCTLA-4–based) therapy. In multivariable analysis, ICI type remained the only variable associated with early SIT use (OR, 12.8; *P* < .001). The IMCES demonstrated areas under the curves (AUCs) of 0.57 (95% CI, 0.50-0.65) for overall and 0.61 (95% CI, 0.53-0.69) for early SIT use.

**Conclusions:**

In patients with ICI colitis, escalation to SIT use was primarily associated with ICI type, with limited additional value of biochemical or endoscopic features. The IMCES showed poor predictive performance in this external validation cohort.

## Teaser

Summary

In this multicenter cohort of 255 patients with checkpoint inhibitor induced colitis, immune checkpoint inhibitor type was the primary determinant of selective immunosuppressive therapy use. Endoscopic features were associated with the use of selective immunosuppressive therapy exclusively in patients with anti-cytotoxic T-lymphocyte–associated antigen 4 (aCTLA-4)–induced colitis, suggesting a context-dependent role for endoscopy.

Key Messages *What is already known?*

Immune checkpoint inhibitor (ICI) colitis is a common immune-related adverse event. Most patients respond to corticosteroids, some require escalation to selective immunosuppressive therapy (SIT; eg, infliximab or vedolizumab). The value of clinical and endoscopic features in predicting need for SIT is uncertain.

*What is new here?*

The ICI type is the dominant determinant of SIT use, while clinical and endoscopic features were of limited added value. Endoscopic features were only associated with SIT use among patients with aCTLA-4–induced colitis.

*How can this study help patient care?*

Added knowledge of differences in the disease course of ICI colitis between ICI types helps clinical assessment and risk stratification. This study indicates that endoscopy may be most informative in patients treated with aCTLA-4-based therapy and highlights the shortcomings of current measurements for risk stratification.

## Introduction

The introduction of immune checkpoint inhibitors (ICIs) has revolutionized the treatment of patients with various types of malignancies.^1^ ICIs are monoclonal antibodies, of which the most commonly used target programmed cell death 1 (PD-1), programmed death-ligand 1 (PD-L1), or cytotoxic T-lymphocyte–associated antigen-4 (CTLA-4).^2^ The expanded oncological indications for ICIs have also increased the prevalence of immune-related adverse events (irAEs). One of the most frequently occurring and serious irAEs is (entero)colitis, which presents with increased bowel frequency and/or abdominal pain. Diarrhea presents in 11% to 35% of patients and the incidence of colitis is 1% to 11%, depending on the type of ICI treatment.^3^ Colitis associated with anti-CTLA-4 (aCTLA-4) monotherapy or combination therapy (aCTLA-4 and aPD-1) typically presents 3 to 11 weeks earlier than aPD-(L)1–induced colitis, with more severe symptoms, and is more frequently treated with immunosuppressive therapy.^3^^,^^4^

Current management of ICI colitis is guided by clinical severity based on the Common Terminology Criteria for Adverse Events (CTCAE) grade.^5^ In patients with CTCAE grade ≥ 2 diarrhea or colitis, ICI treatment is generally withheld and systemic corticosteroids are initiated.^6^^,^^7^ Escalation to selective immunosuppressive therapy (SIT), including infliximab and vedolizumab, is necessary in patients with an inadequate response to corticosteroids.^6^ Both high corticosteroid peak dose and SIT use have been associated with decreased overall survival,^8–11^ a finding that highlights the need for early identification of patients in need of treatment escalation. Previous studies have shown that clinical severity, as defined by CTCAE grade, is not a reliable tool to identify patients who require SIT.^12–14^

Consequently, there is a need for objective parameters to guide risk stratification and treatment decisions in patients with ICI colitis. Earlier studies have demonstrated an association between ulceration found on endoscopy and SIT use, findings suggesting that endoscopy may help in the prediction of disease course.^12^^,^^15^^,^^16^ However, this association has been identified primarily in older patient cohorts treated when aCTLA4 monotherapy was more commonly prescribed, while aPD-(L)1 therapy and combination therapy are currently given more frequently. Prescribing strategies and clinical guidelines have evolved ever since,^17^ raising the question of whether this association holds true in current clinical practice. Compared to aPD-(L)1–induced colitis, aCTLA-4–induced colitis has been reported to exhibit a distinct clinical and endoscopic pattern with more severe endoscopic findings and a higher frequency of ulceration.^18^ How these differences in endoscopic appearance relate to the prediction of ICI colitis disease course and need for SIT among different ICI types remains unclear, as prior studies have not accounted for ICI-specific effects.

Recently, a new endoscopic scoring tool has been proposed to predict the need for SIT in patients with ICI colitis. The immune-mediated colitis endoscopic score (IMCES) is a composite of 10 endoscopic features that has shown promising discriminative performance.^19^ The original study and the follow-up internal validation study in the same center reported specificities ranging from 82% to 92%.^19^^,^^20^ However, the IMCES has not yet been externally validated and its discriminative performance has not been evaluated separately for aPD-(L)1– and aCTLA-4–induced colitis.

In this study, we aimed to identify clinical and endoscopic factors associated with the need for SIT in patients with histologically confirmed ICI colitis. We also sought to evaluate whether these associations differ by ICI type. Furthermore, we aimed to externally validate the newly developed IMCES endoscopy score and to assess its discriminative performance stratified by ICI type.

## Materials and methods

### Study design

We performed a retrospective cohort study in 2 university medical centers in the Netherlands. All included patients were aged ≥ 18 years and (1) received ICIs for solid tumors during the period of January 2014-December 2024; (2) developed diarrhea and/or colitis; (3) underwent endoscopy with biopsies; and (4) had pathology reports that indicated an ICI diagnosis. Patients were excluded if: (1) endoscopy was performed >5 days after treatment for ICI colitis was initiated, (2) endoscopic images could not be retrieved for review, or (3) SIT was prescribed as the first treatment.

### Data collection

Electronic medical records were reviewed to collect clinical data, including demographics, medical history, oncologic history, laboratory results, endoscopic and histopathological evaluation results, clinical course of colitis, colitis treatment, and overall survival.

### Endoscopy review

Endoscopic images were reviewed by 2 experts in IBD endoscopy, who were blinded to the endoscopy reports and clinical outcomes (M.V. and F.v.S.). All images were scored according to the Mayo Endoscopic Score (MES)^21^  and the previously published, newly proposed IMCES.^19^ The IMCES includes 10 endoscopic features, each scored as 1 point when present. The following features were assessed: ulceration, large ulcers, deep ulcers,  ≥ 3 ulcers, erythema, loss of vasculature, friability, erosions, exudate, and extensive inflammation. Extensive inflammation was defined as ≥2 segments showing the abovementioned features.^19^ In case of sigmoidoscopies, both the rectum and sigmoid had to show signs of inflammation. Uncertain cases were re-evaluated until consensus was reached.

### Definitions and endpoints

Patients were analyzed according to the most recent ICI type diagnosed prior to the onset of ICI colitis. Treatment was categorized as either aPD-(L)1 monotherapy, including aPD-1– and aPD-L1–therapy, or aCTLA-4–based therapy, which included aCTLA-4 monotherapy and combination therapy. Follow-up was defined as the period from the date of ICI colitis diagnosis according to endoscopy and/or the start of first treatment, whichever occurred first, until the end of colitis treatment or death, whichever occurred first. Severities of diarrhea and colitis were determined according to the CTCAE grade, version 5.^5^

The primary endpoint was SIT use at any timepoint during follow-up. The secondary endpoint was the need for early SIT use, defined as SIT use within 3 weeks after starting the first colitis treatment.

### Statistical analysis

Continuous variables were presented as means with SDs or medians with IQRs depending on the normality of the distribution. Comparison of continuous variables was done using *t*-tests. Categorial variables were presented as percentages and compared by use of the chi-square test. Follow-up time among treated patients was evaluated with the reverse Kaplan-Meier method, in which deaths occurring before treatment completion were censored. Univariate logistic regression was performed to identify factors associated with SIT use and early SIT use. A *P* value < .05 was considered statistically significant. Analyses were performed on complete cases for each variable. Patients with missing data were excluded from the respective analyses. Variables were selected for multivariate analysis based on clinical relevance and prior literature, and univariable analysis was used to support selection. Likelihood ratio testing (LRT) was used to compare nested models to assess the added significance of individual covariates. All tests were 2-sided.

The discriminative performance of variables associated with SIT use was evaluated by analysis of the receiver operating characteristic (ROC) and corresponding area under the curve (AUC). Comparison of AUCs between subgroups were done using the DeLong test for independent ROC curves. Sensitivity, specificity, positive predictive value (PPV), and negative predictive value (NPV) for (early) SIT use were calculated for IMCES cutoff scores of 4 and 5. These cutoff scores were chosen in accordance with the study by Wang et al.^19^ Analyses were performed using R version 4.4.0.

## Results

### Patient characteristics

Of 279 patients identified with histologically confirmed ICI colitis, 255 were eligible for study inclusion (Figure S1). Patient characteristics are summarized in Table 1. Patients had a median age of 66 years (IQR, 57-73 years) and 51% received aPD-(L)1 monotherapy. In the group receiving aCTLA-4 based therapy, 88% of patients were treated with combination therapy. Among patients receiving treatment for ICI colitis, median follow-up was 103 days (95% CI, 92-122). Melanoma was the most common tumor type (53%). Patients on aCTLA-4 based therapy had received prior ICI therapy more frequently than patients treated with aPD-(L)1 therapy (*n* = 31 (25%) vs *n* = 6 (5%), *P* < .001), whereas patients on aPD-(L)1 therapy more frequently had prior treatment with chemotherapy or chemoradiotherapy (*P* < .001).

**Table 1:** Baseline characteristics of included patients.

	Total	PD-(L)1 therapy	CTLA-4 monotherapy or combination therapy
	*n* = 255	*n* = 131	*n* = 124
**Patient characteristics**			
**Age, median (IQR) y**	66 (57-73)	69 (61-74)	64 (54-72)
**Sex, female**	112 (44%)	60 (46%)	52 (42%)
**Tumor type**			
** Melanoma**	136 (53%)	35 (27%)	101 (81%)
** Non-small cell lung cancer**	53 (21%)	47 (36%)	6 (4.8%)
** Renal cell carcinoma**	20 (7.8%)	6 (4.6%)	14 (11%)
** Other**	46 (18%)	44 (33%)	3 (2.4%)
**Immunotherapy type**			
** aPD(L1)-therapy**	131 (51%)	100%	0 (0%)
** aCTLA-4 monotherapy**	15 (5.9%)	0 (0%)	15 (12%)
** Combination therapy**	109 (43%)	0 (0%)	109 (87%)
**Prior other treatments**			
** Immunotherapy**	37 (15%)	6 (4.6%)	31 (25%)
** Targeted therapy**	7 (2.7%)	7 (5.3%)	0 (0%)
** Chemotherapy**	27 (11%)	25 (19%)	2 (1.6%)
** Chemoradiotherapy**	24 (9.4%)	22 (17%)	2 (1.6%)
** BRAF-MEK inhibition**	26 (10%)	3 (2.3%)	23 (19%)
**Clinical features of ICI colitis**			
**Follow-up, median (IQR) d**	62 (17-115)	82 (31-135)	50 (12-91)
**Time to onset after initiation of ICI, median (IQR) d**	57 (25-188)	154 (70-325)	33 (20-52)
**CTCAE grade at onset**			
** Grade 1**	43 (17%)	25 (19%)	18 (15%)
** Grade 2**	116 (45%)	67 (51%)	49 (40%)
** Grade 3**	94 (37%)	37 (28%)	57 (46%)
** Grade 4**	2 (0.8%)	2 (1.5%)	0 (0%)
** Grade 5**	0 (0%)	0 (0%)	0 (0%)
**Hospitalized**	166 (65%)	63 (48%)	103 (83%)
**Treatment of ICI colitis**			
**Time to therapy, median (IQR) d**	11 (6-21)	17 (9-33)	8 (5-14)
**Treatment of ICI colitis**			
** No immunosuppressive therapy**	28 (11%)	22 (17%)	6 (4.8%)
** Exclusive topical therapy**	17 (6.7%)	14 (11%)	3 (2.4%)
** Systemic corticosteroids**	114 (44%)	58 (44%)	55 (44%)
** SIT**	98 (38%)	37 (28%)	60 (48%)
** Early SIT use**	59 (23%)	12 (9.2%)	47 (38%)
**Duration of systemic steroids, median (IQR) d**	85 (58-140)	91 (62-151)	78 (57-127)
**Cumulative dose of systemic steroids, median (IQR) g**	3.2 (2.0-4.8)	2.9 (2.0-4.2)	3.3 (2.1-5.1)
**Time to SIT initiation, median (IQR) d**	15 (5-52)	60 (17-102)	7 (5-20)

Abbreviations: aCTLA-4, anti-cytotoxic T-lymphocyte-associated antigen 4; aPD-(L)1, anti-programmed cell death(-ligand) 1; CTCAE, common terminology criteria for adverse events; ICI, immune checkpoint inhibitor; SIT, selective immunosuppressive therapy.

### Clinical characteristics of ICI colitis

Most patients presented with a CTCAE grade 2 diarrhea/colitis (*n* = 116, 45%). The CTCAE grade for diarrhea/colitis at onset was higher for patients who received aCTLA-4–based therapy (grade 2, *n* = 49 (40%); grade 3, *n* = 57 (46%)) than for patients on aPD-(L)1 therapy (grade 2, *n* = 67 (51%), or grade 3, *n* = 37 (28%), *P* = .014). Time from ICI initiation to onset of symptoms differed significantly between the 2 ICI groups (aPD-(L)1 therapy, 154 days; IQR, 70-325 days; aCTLA-4–based therapy, 33 days; IQR, 20-52 days; *P* < .001), as well as the time from symptom onset to initiation of colitis treatment (aPD-[L]1 therapy, 17 days; IQR, 9-33 days; aCTLA-4-based therapy 8 days; IQR, 5-14 days; *P* < .001). Overall, 166 patients (65%) were hospitalized (aPD-[L]1–treated patients, *n* = 63 (48%); aCTLA-4–treated patients, *n* = 103 (83%), *P* < .001).

### Biochemical characteristics

Laboratory values collected around the diagnosis date (median, 0 days; IQR, −1 to 0 days) showed a median CRP level of 30 mg/L (IQR, 8-71 mg/L) and albumin level of 37.0 g/L (IQR, 32.0-40.2 mg/L). The CRP level was significantly higher for patients with an aCTLA-4–based induced colitis (51 mg/L, IQR, 28-102 mg/L), compared to patients with aPD-(L)1–induced colitis (12 mg/L, IQR 3-35 mg/L, *P* < .001). Albumin levels also differed significantly (aCTLA-4, 35.0 g/L; vs aPD-[L]1 therapy, 39.0 g/L; *P* = .005). Calprotectin values did not differ significantly (*P* = .3). All collected laboratory values are summarized in Table S1.

### Endoscopic characteristics

Endoscopic evaluation was most often performed by sigmoidoscopy (*n* = 161, 63%). The distribution of endoscopic Mayo scores differed significantly between patients on aPD-(L)1 therapy and patients on aCTLA-4 based therapy (Table 2, *P* value < .001). The IMCES scores did not differ significantly between the groups (*P* = .056). Endoscopic features are summarized in Table 2. Loss of vasculature and erythema were most often observed (*n* = 199, 78%). Patients on aCTLA-4–based therapy more often displayed extensive inflammation (*n* = 67, 54% vs *n* = 45, 34%; *P* = .002) and erosions (*n* = 49, 40% vs *n* = 36, 27%; *P* = .042) compared to patients on aPD-(L)1 therapy. Ulcerations were present in 15% (*n* = 39) of the total cohort.

**Table 2:** Endoscopic features of patients diagnosed with ICI colitis.

	Total	aPD-(L)1 therapy	Combination or aCTLA-4 monotherapy
	*n* = 255	*n* = 131	*n* = 124
**Time to endoscopy, median (IQR) d**	11 (6-19)	14 (8-29)	8 (5-14)
**Type of endoscopy**			
** Sigmoidoscopy**	161 (63%)	76 (58%)	85 (69%)
** Colonoscopy**	94 (37%)	55 (42%)	39 (31%)
**Endoscopic Mayo score**			
** Mayo 0**	44 (17%)	28 (21%)	16 (13%)
** Mayo 1**	90 (35%)	57 (44%)	33 (27%)
** Mayo 2**	67 (26%)	20 (15%)	47 (38%)
** Mayo 3**	54 (21%)	26 (20%)	28 (23%)
**IMCES, median, (IQR)**	3 (2-5)	3 (2-4)	3 (2-5)
**Frequency of IMCES endoscopic features**			
** Loss of vasculature**	199 (78%)	106 (81%)	93 (75%)
** Erythema**	199 (78%)	101 (77%)	98 (79%)
** Extensive inflammation**	112 (44%)	45 (34%)	67 (54%)
** Erosions**	85 (33%)	36 (27%)	49 (40%)
** Friability**	81 (32%)	36 (27%)	45 (36%)
** Exudate**	50 (20%)	23 (18%)	27 (22%)
** Ulceration**	39 (15%)	20 (15%)	19 (15%)
** Multiple ulcers**	23 (9.0%)	11 (8.4%)	12 (9.7%)
** Large ulcers**	8 (3.1%)	2 (1.5%)	6 (4.8%)
** Deep ulcers**	3 (1.2%)	0 (0%)	3 (2.4%)
**No IMCES endoscopic features**	32 (13%)	19 (15%)	13 (10%)

Abbreviations: aPD(L)1, anti–programmed cell death(-ligand) 1; aCTLA-4, anti–cytotoxic T-lymphocyte–associated antigen 4; CTCAE, common terminology criteria for adverse events; ICI, immune checkpoint inhibitor; IMCES, immune-mediated colitis endoscopic score.

### Histopathology

Inflammation patterns were based on histopathology reports, with acute–active inflammation observed in 82 patients (32%), chronic inflammation in 20 patients (8%), and a combination of both types in 103 patients (40%). Histologic features were variably reported in pathology reports. Among cases in which features were explicitly reported, increased apoptotic bodies were present in 194 of 215 cases, neutrophils in lamina propria in 102 of 105 cases, and epithelial neutrophils in 117 of 130 cases. Granulomas were reported in 9 of 111 cases.

### Frequency of SIT use

Overall, 98 patients (38%) were treated with SIT, after initial treatment with topical and/or systemic corticosteroids. SIT was initiated earlier in patients treated with aCTLA-4 based therapy (median 7 vs 60 days from initiation of any therapy, *P* < .001). Infliximab was used as the first SIT in 76% (*n* = 74) of patients treated with SIT, and vedolizumab in 22% of patients (*n* = 22). Multiple SITs were required in 15% (*n* = 15) of patients for treatment of ICI colitis. Of all patients receiving SIT, 85 (87%) were in full remission and 10 (10%) were in partial remission at the end of follow-up. Treatment sequences of the total cohort are illustrated in Figure 1.

**Figure 1:**
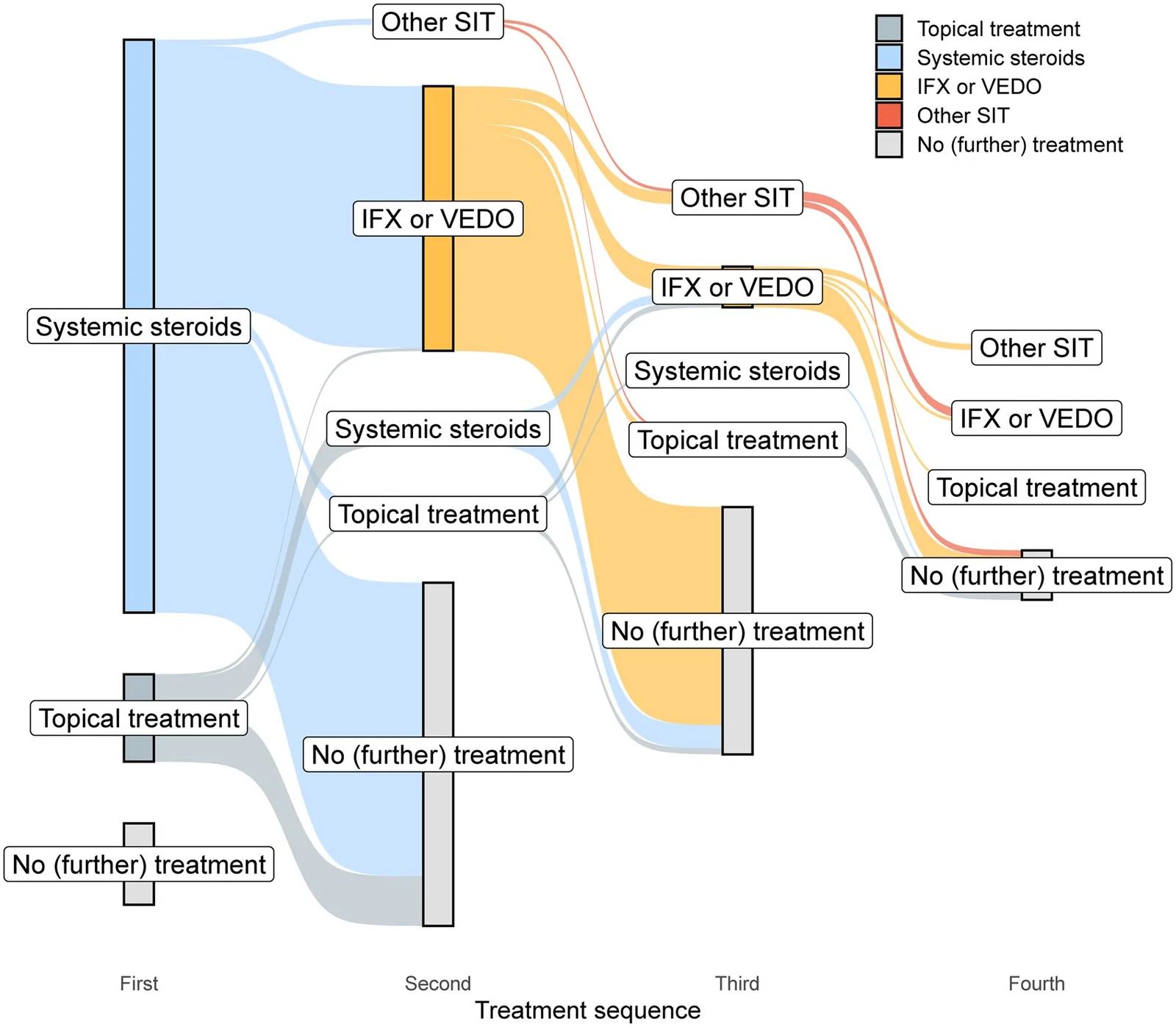
Sankey diagram illustrating treatment escalation of patients receiving therapy for ICI colitis, from first to fourth line of therapy. The width of the flows represents the number of patients progressing through each treatment sequence. Most patients were first treated with systemic steroids. The second treatment line shows that many patients required no additional therapy; among those who did, the most common subsequent treatments were infliximab or vedolizumab. Abbreviations: SIT, selective immunosuppressive therapy; IFX, infliximab; VEDO, vedolizumab.

### Factors associated with SIT use

Factors associated with overall and early SIT use are shown in Tables 3 and 4. Clinical associations with both early and overall SIT use were hospitalization (early SIT OR, 14.6; 95% CI, 4.42-48.2), time from ICI initiation to onset of complaints (early SIT per week, OR, 0.95; 95% CI, 0.93-0.98). Biochemical parameters were primarily associated with early SIT use: increased CRP level (log-transformed OR, 1.41; 95% CI, 1.11-1.80) and decreased albumin level (OR, 0.92; 95% CI 0.87-0.99). Endoscopic features were only associated with early SIT use and not with overall SIT use during follow-up in the total cohort. In the subgroup analysis of patients undergoing aCTLA-4 based treatment, only the presence of erythema (OR, 4.09; 95% CI, 1.51-11.1) and exudate (OR, 3.24; 95% CI, 1.29-8.13) were associated with overall SIT use during follow-up, whereas more features showed association with early SIT use (Table 4). Among patients treated with aPD-(L)1 therapy, no endoscopic features were associated with early or overall SIT use. Ulcerations were not significantly associated with SIT use in any of the analyses.

**Table 3:** Factors associated with SIT use.

	Total			aPD-(L)1 therapy			Combination or aCTLA-4 monotherapy		
	OR	95% CI	*P* value	OR	95% CI	*P* value	OR	95% CI	*P* value
**Age**	0.98	0.96-1.00	0.061	0.98	0.94-1.01	0.195	0.99	0.96-1.02	0.474
**Sex, female**	1.83	1.10-3.05	0.021	2.32	1.07-5.02	0.034	1.67	0.81-3.42	0.163
**Hospitalization**	4.04	2.19-7.43	<0.001	3.89	1.72-8.79	0.001	2.76	0.99-7.66	0.052
**Time from initiation of ICI to onset of complaints, wk**	0.97	0.96-0.99	<0.001	0.98	0.96-1.00	0.021	0.96	0.91-1.01	0.143
**Time from onset to therapy, wk**	0.85	0.75-0.97	0.013	0.90	0.78-1.03	0.126	0.82	0.59-1.15	0.250
**Laboratory values**									
**Hemoglobin**	1.13	0.91-1.39	0.274	1.03	0.74-1.44	0.850	1.15	0.87-1.53	0.326
**Leucocytes**	0.98	0.93-1.05	0.614	0.91	0.80-1.04	0.172	1.02	0.92-1.14	0.643
**Albumin**	1.01	0.95-1.06	0.853	1.06	0.97-1.15	0.214	0.97	0.89-1.06	0.503
**CRP (log transformed)**	1.30	1.06-1.60	0.011	1.17	0.88-1.58	0.283	1.10	0.76-1.61	0.605
**Calprotectin (log transformed)**	1.01	0.83-1.24	0.899	1.09	0.82-1.45	0.556	0.90	0.67-1.21	0.473
**Endoscopic features**									
**IMCES, continuous**	1.13	1.00-1.27	0.057	0.93	0.77-1.13	0.487	1.28	1.07-1.53	0.008
**IMCES, cutoff 4**	1.45	0.87-2.43	0.159	0.59	0.25-1.40	0.232	2.33	1.13-4.80	0.022
**Features of IMCES**									
** Loss of vasculature**	1.16	0.63-2.15	0.636	0.67	0.27-1.68	0.394	2.02	0.87-4.69	0.100
** Erythema**	1.42	0.76-2.66	0.275	0.52	0.22-1.22	0.134	4.09	1.51-11.1	0.006
** Extensive inflammation**	1.22	0.73-2.03	0.443	1.40	0.63-2.99	0.431	0.83	0.41-1.69	0.609
** Erosions**	1.38	0.81-2.34	0.237	0.62	0.25-1.52	0.295	2.06	0.99-4.28	0.053
** Friability**	1.68	0.98-2.87	0.058	1.11	0.48-2.56	0.810	2.09	0.99-4.41	0.052
** Exudate**	1.81	0.97-3.37	0.063	0.84	0.30-2.32	0.734	3.24	1.29-8.13	0.012
** Ulceration**	1.46	0.73-2.90	0.283	0.79	0.26-2.35	0.668	2.67	0.94-7.57	0.064
** Multiple ulcers**	1.53	0.65-3.61	0.334	0.91	0.23-3.63	0.895	2.31	0.66-8.11	0.192
** Large ulcers**	0.96	0.22-4.11	0.956	NA	NA	NA	1.07	0.21-5.52	0.935
** Deep ulcers**	3.25	0.29-36.3	0.339	NA	NA	NA	2.17	0.19-24.6	0.531
**Endoscopic Mayo score**									
** Mayo 0**	ref			ref			ref		
** Mayo 1**	1.19	0.55-2.61	0.659	1.06	0.39-2.88	0.905	1.43	0.40-5.08	0.580
** Mayo 2**	2.18	0.97-4.88	0.058	0.83	0.23-3.07	0.784	2.97	0.89-9.91	0.077
** Mayo 3**	1.77	0.76-4.11	0.185	1.11	0.35-3.58	0.860	2.54	0.70-9.24	0.158

Abbreviations: aCTLA-4, anti-cytotoxic T-lymphocyte-associated antigen 4; aPD-(L)1, anti-programmed cell death (ligand) 1; CRP, C-reactive protein; ICI, immune checkpoint inhibitor; IMCES, immune-mediated colitis endoscopic score; NA, not applicable.

**Table 4:** Factors associated with early SIT use.

	Total			aPD-(L)1 therapy			Combination or aCTLA-4 monotherapy		
	OR	95% CI	*P* value	OR	95% CI	*P* value	OR	95% CI	*P* value
**Age**	0.98	0.95-1.01	0.121	0.98	0.92-1.02	0.252	1.00	0.97-1.03	.910
**Sex, female**	1.72	0.96-3.09	0.070	4.00	1.03-15.5	0.045	1.59	0.76-3.31	.218
**Hospitalization**	14.6	4.42-48.2	<0.001	NA	NA	NA	4.47	1.24-16.1	.022
**Time from initiation of ICI to onset of complaints, wk**	0.95	0.93-0.98	<0.001	0.98	0.95-1.01	0.197	0.95	0.89-1.02	.151
**Time from onset to therapy, weeks**	0.72	0.58-0.89	0.003	0.88	0.69-1.12	0.303	0.73	0.51-1.06	.102
**Laboratory values**									
**Hemoglobin**	1.09	0.86-1.39	0.455	0.79	0.49-1.28	0.342	1.15	0.86-1.54	.352
**Leucocytes**	1.02	0.95-1.08	0.631	0.97	0.82-1.14	0.678	1.02	0.92-1.14	.664
**Albumin**	0.92	0.87-0.99	0.020	0.88	0.77-1.00	0.043	0.97	0.89-1.05	.428
**CRP (log transformed)**	1.41	1.11-1.80	0.005	1.27	0.83-1.94	0.280	1.02	0.70-1.50	.912
**Calprotectin (log transformed)**	1.07	0.85-1.35	0.561	0.99	0.65-1.50	0.959	1.02	0.76-1.37	.901
**Endoscopic features**									
**IMCES, continuous**	1.19	1.03-1.36	0.016	0.96	0.71-1.30	0.784	1.24	1.03-1.48	.021
**IMCES, cut off 4**	1.95	1.08-3.51	0.027	0.41	0.09-1.96	0.265	2.44	1.16-5.13	.019
**Features of IMCES**									
** Loss of vasculature**	2.07	0.92-4.66	0.080	2.78	0.34-22.6	0.339	2.59	1.01-6.60	.047
** Erythema**	3.03	1.23-7.46	0.016	1.54	0.32-7.44	0.592	4.30	1.38-13.4	.012
** Extensive inflammation**	1.10	0.61-1.98	0.745	0.95	0.27-3.35	0.938	0.72	0.35-1.49	.374
** Erosions**	1.83	1.01-3.33	0.048	0.50	0.10-2.40	0.387	2.17	1.03-4.57	.041
** Friability**	1.52	0.83-2.79	0.176	0.87	0.22-3.41	0.840	1.54	0.73-3.26	.258
** Exudate**	2.00	1.02-3.93	0.045	0.40	0.05-3.27	0.393	3.10	1.29-7.45	.012
** Ulceration**	1.60	0.75-3.39	0.222	0.48	0.06-3.93	0.492	2.64	0.97-7.13	.057
** Multiple ulcers**	1.51	0.59-3.88	0.387	0.99	0.12-8.48	0.993	1.73	0.52-5.72	.368
** Large ulcers**	1.11	0.22-5.66	0.899	NA	NA	NA	0.81	0.14-4.61	.813
** Deep ulcers**	1.67	0.15-18.8	0.677	NA	NA	NA	0.82	0.07-9.25	.869
**Endoscopic Mayo score**									
** Mayo 0**	ref			ref			ref		
** Mayo 1**	1.56	0.53-4.61	0.421	3.78	0.44-32.4	0.225	0.96	0.24-3.83	.954
** Mayo 2**	4.64	1.62-13.33	0.004	4.76	0.46-49.6	0.192	2.64	0.74-9.39	.134
** Mayo 3**	2.73	0.90-8.30	0.077	1.08	0.06-18.2	0.957	2.60	0.67-10.1	.166

Abbreviations: aPD-(L)1, anti-programmed cell death–ligand 1; aCTLA-4, anti-cytotoxic T-lymphocyte-associated antigen 4; ; CRP, C-reactive protein; ICI, immune checkpoint inhibitor; IMCES, immune-mediated colitis endoscopic score.

In multivariate logistic regression including sex, CRP, albumin level, endoscopic Mayo score, and ICI type, only ICI type (aCTLA-4 based therapy as opposed to aPD-[L]1 therapy; OR, 12.8; *P* < .001) and a Mayo score of 2 (OR, 11.2; *P* = .035) were significantly associated with early SIT use (Table 5). The LRT demonstrated that inclusion of ICI type significantly improved model fit (*P* < .001), whereas stepwise removal of other covariates, including the Mayo score, did not significantly worsen model performance.

**Table 5:** Multivariate logistic regression for early SIT use.

	OR	95% CI	*P* value
**Sex, ref = male**	1.88	0.67-5.19	.215
**ICI type, ref = aPD-(L)1 therapy**	12.3	3.80-51.5	<.001
**CRP (log transformed)**	0.81	0.53-1.23	.319
**Albumin**	0.92	0.83-1.02	.127
**Endoscopic Mayo score**			
** Mayo 0**	ref		
** Mayo 1**	4.54	0.58-97.8	.207
** Mayo 2**	11.2	1.69-228	.035
** Mayo 3**	7.52	0.70-5.19	.086

Abbreviations: CRP, C-reactive protein; ICI, immune checkpoint inhibitor; aPD-(L)1, anti-programmed cell death(-ligand) 1.

### Discriminative performance of clinical and endoscopic variables

The ROC analyses of CRP, albumin level, and Mayo score demonstrated AUCs ranging from 0.61 to 0.64 for predicting early SIT use (Figure S2). Combining CRP and albumin with the endoscopic Mayo score did not improve the AUC significantly compared to the performance of individual variables.

The AUC of the IMCES for predicting SIT use was low and comparable for overall SIT use and early SIT use (Table 6, respectively, 0.57; 95% CI, 0.50-0.65; 0.61, 95% CI, 0.53-0.69). Sensitivity and specificity for predicting overall SIT use were 44% and 65% at a cutoff of 4, respectively. Subgroup analyses among patients treated with aPD-(L)1 therapy and aCTLA-4 based therapy revealed no significant difference between these groups for predicting early SIT use (*P* = .2).

**Table 6:** Performance of IMCES in predicting SIT use.

Population	SIT outcome	AUC (95% CI)	Cutoff	Sensitivity	Specificity	PPV	NPV
**Total cohort**	SIT use during follow up	0.57 (0.50 - 0.65)	4	0.44	0.65	0.44	0.66
			5	0.32	0.79	0.48	0.65
	Early SIT use	0.61 (0.53 - 0.69)	4	0.51	0.65	0.31	0.82
			5	0.36	0.78	0.33	0.80
**aPD-(L)1 therapy**	SIT use during follow up	0.47 (0.36 - 0.58)	4	0.24	0.66	0.22	0.69
			5	0.19	0.78	0.25	0.71
	Early SIT use	0.53 (0.38 - 0.68)	4	0.92	0.23	0.11	0.96
			5	0.92	0.13	0.10	0.94
**aCTLA-4 monotherapy or combination therapy**	SIT use during follow up	0.64 (0.55 - 0.74)	4	0.57	0.64	0.66	0.59
			5	0.40	0.81	0.66	0.59
	Early SIT use	0.65 (0.55 - 0.74)	4	0.60	0.62	0.49	0.72
			5	0.43	0.79	0.55	0.69

Abbreviations: IMCES, Immune-Mediated colitis Endoscopic Score; SIT, selective immunosuppressive therapy; AUC, area under the curve; PPV, positive predictive value; NPV, negative predictive value; aPD-(L)1, anti-programmed cell death(-ligand) 1; aCTLA-4, anti-cytotoxic T-lymphocyte-associated antigen 4

## Discussion

In this 2-center, retrospective cohort study, we evaluated clinical and endoscopic factors associated with SIT use in patients with ICI colitis. Although many retrospective studies to date have described the clinical, endoscopic, and histopathological features of ICI-induced colitis, this is the first study to systematically assess associations of these features with SIT use stratified by ICI type. Although several clinical variables and endoscopic features were associated with SIT use and early SIT use in univariate analysis, the use of aCTLA-4–based therapy emerged as the strongest association with (early) SIT use in multivariable analysis. In line with this finding, endoscopic features were only associated with SIT use among patients treated with aCTLA-4–based therapy. The recently developed IMCES demonstrated poor discriminative performance in this external validation cohort.

This study shows the ICI type to be the most dominant determinant of SIT use. Given the importance of ICI type for SIT initiation, we examined whether variables provided additional prognostic value within individual ICI classes. Because the disease course and timing of SIT initiation can differ substantially between ICI types, both early SIT use and overall SIT use were evaluated. Early SIT use as an endpoint indicates patients that develop a more severe, potentially steroid-refractory disease course and who need early treatment escalation.

In this study several ICI-dependent associations between endoscopic features and SIT use were found. It is well established that aCTLA-4–induced colitis typically presents earlier, is clinically more severe, and more frequently requires SIT than aPD-(L)1–induced colitis.^3^^,^^4^ In a recent study, Shatila et al. found that aside from the earlier and more severe clinical presentation, aCTLA-4–based treatment was associated with more ulcerative lesions on endoscopy.^18^ Our results indicate that this difference also extends to the associations of endoscopic features with SIT use. Whereas several endoscopic features were associated with early and overall SIT use among patients treated with aCTLA-4–based therapy, no such associations were observed in the aPD-(L)1 subgroup, and both the Mayo endoscopic score and IMCES demonstrated a trend toward a poorer discriminative performance in this group. This difference in associations suggests that the clinical utility of endoscopy in ICI colitis may vary according to ICI type.

Overall, our results indicate that endoscopic assessment alone is insufficient to implement as a predictor for SIT use, as established by the limited results of both the IMCES and endoscopic Mayo score in ROC analysis. Erythema and exudate showed the strongest association with SIT use among patients treated with aCTLA-4–based therapy. However, erythema is a nonspecific and commonly observed feature, which limits its discriminative value. Notably, ulcerative lesions were not associated with SIT use in any analysis, contrasting with older studies.^12^^,^^15^^,^^16^ These discrepancies may reflect a change in management of ICI colitis, eg, earlier and more consistently applied endoscopic assessment, or a shift in ICI prescription strategy influencing the endoscopic phenotype.

These results warrant reconsideration of endoscopy as prognostic tool in ICI colitis. The IMCES was recently developed to guide treatment escalation based on endoscopic findings in patients with ICI colitis.^19^ In contrast to the high discriminative performance reported in the derivation and internal validation studies, the IMCES demonstrated a substantially poorer performance in our cohort, even though our population shows a similar proportion of aPD-(L)1 therapy, distribution in time to onset of complaints, and SIT initiation rate.^19^

A key difference between our studies lies in the method of endoscopic assessment. The IMCES was derived from endoscopy reports,^19^^,^^20^ whereas in our study all evaluations were conducted on original endoscopy images. Direct review of images likely increased detection of subtle inflammatory features, such as erythema and loss of vasculature, which were observed at higher frequencies in our study. In addition, endoscopy was performed slightly earlier in our cohort (11 days [IQR, 6-19] vs 15 days (IQR, 9-38]). It is unknown whether such a difference affects endoscopic disease severity, since data on the longitudinal endoscopic evaluation of ICI colitis were lacking. However, a tool like the IMCES should demonstrate reliable performance at an early stage in the disease course to be able to support clinical decision-making regarding escalation to SIT. Finally, the limited performance of the IMCES may also relate to the construction of the score itself, as previously elaborated by Bergqvist et al.^22^ Taken together, these limitations and the poor performance as shown in this study indicate that the IMCES, in its current form, is not suitable for use in clinical practice.

Elevated CRP and decreased albumin were associated with early SIT use in univariate analysis. This finding corresponds with measurements of disease activity known in IBD.^23–26^ However, these associations were not retained in multivariable analysis and laboratory values differed significantly between patients treated with aCTLA-4 based therapy and aPD-(L)1 therapy. This suggests that the moderate effect in the overall cohort is driven by difference in these markers between ICI type. A previous study did not find any association between CRP, albumin or hemoglobin with treatment outcomes and pointed to the difference in age and comorbidities compared to the typical IBD population.^14^ Combining endoscopy and biochemical parameters did not significantly improve discriminative performance. Overall, these results underline the finding that currently available clinical and endoscopic variables are of limited value in identifying patients in need of future SIT.

Key strengths of this study include the use of a carefully selected cohort from 2 tertiary centers with extensive experience in ICI treatment and toxicity management. All included patients had endoscopically and histologically confirmed ICI-induced colitis, in contrast to many prior studies in which diagnosis definitions relied partially on CTCAE grading or steroid exposure. Such approaches may result in heterogenous cohorts and risk misdiagnosis. In addition, all endoscopic findings were assessed through direct review of images by an IBD-specialized gastroenterologist, ensuring a high level of accuracy in endoscopic severity assessment.

Several limitations should also be acknowledged. The retrospective design restricts insight into causal interference of analyzed features. Furthermore, the frequent use of sigmoidoscopies may have led to an underestimation of disease severity of patients with right-sided inflammation, which may account for 3%-8% of cases.^16^^,^^27^ In addition, death may have acted as a competing event for initiation of SIT, as analyses were based on logistic regression rather than time-to-event methods. However, cumulative incidence functions of SIT use and death events demonstrated that SIT use occurred substantially earlier than death in our cohort (Figure S3), suggesting that the impact of this competing risk is likely limited. Last, changes in management strategies for ICI colitis over time are difficult to capture in retrospective studies and may have influenced both endoscopic findings and treatment decisions. A separate analysis of aCTLA-4 monotherapy was not feasible due to the relatively small number of patients on aCTLA-4 monotherapy.

In conclusion, in this study on ICI colitis, escalation to SIT was necessary in 38% of patients and was primarily driven by ICI type, with limited predictive value of biochemical and endoscopic parameters. The endoscopic scoring tool IMCES showed a poor performance in predicting SIT use. These findings highlight the importance of prospective studies employing structured and detailed data collection as well as the incorporation of immunological and molecular biomarkers to improve prediction of ICI colitis outcomes.

## Supplementary Material

izag104_Supplementary_Data

## Data Availability

The data used for this study are available from the corresponding author upon reasonable request.
