# Supplementary material for: Clinical and endoscopic features of checkpoint inhibitor–induced colitis and their association with selective immunosuppressive therapy use
Source: Inflamm Bowel Dis. 2026 Aug 21;32(11):2118–28. doi: 10.1093/ibd/izag104 (PMC13722126; doi:10.1093/ibd/izag104)
Supplement: Supplementary file 1 [file izag104_supplementary_data.docx]

**Supplementary**

| Supplementary Table 1: Laboratory values collected at baseline | | | | | | | | | | | | | |
| --- | --- | --- | --- | --- | --- | --- | --- | --- | --- | --- | --- | --- | --- |
|  |  |  |  |  |  |  |  |  |  |  |  |  |  |
|  |  |  | Total | | |  | aPD-(L)1 therapy | | |  | aCTLA-4 monotherapy or combination therapy | | |
|  |  |  | N = 255 | | |  | N = 131 | | |  | N = 124 | | |
|  | Unit of measurement |  | n | Median | IQR |  | n | Median | IQR |  | n | Median | IQR |
| Hemoglobin | mmol/L |  | 214 | 8.2 | 7.2 - 9.0 |  | 101 | 8.1 | 7.2 - 8.7 |  | 113 | 8.3 | 7.3 - 9.2 |
| Leucocytes | *10^9/L |  | 211 | 8.4 | 6.3 - 10.8 |  | 100 | 7.8 | 6.1 - 9.8 |  | 111 | 8.9 | 6.8 - 11.0 |
| Platelets | *10^3 per mm^3 |  | 209 | 279 | 228 - 357 |  | 99 | 257 | 206 - 331 |  | 110 | 292 | 250 - 390 |
| Creatinin | µmol/L |  | 221 | 75 | 62 - 93 |  | 106 | 76 | 64 - 100 |  | 115 | 74 | 61 - 90 |
| Albumin | g/L |  | 156 | 37.0 | 32.0 - 40.2 |  | 73 | 39.0 | 34.4 - 41.5 |  | 83 | 35.0 | 31.3 - 39.0 |
| CRP | mg/L |  | 197 | 30 | 8 - 71 |  | 96 | 12 | 3 - 35 |  | 101 | 51 | 28 - 102 |
| Faecal calprotectin | µg/g |  | 126 | 469 | 147 - 1460 |  | 63 | 452 | 110 - 1460 |  | 63 | 514 | 174 - 1590 |
| Abbreviations: aPD-(L)1, anti-programmed cell death(-ligand) 1; aCTLA-4, anti-cytotoxic T-lymphocyte-associated antigen 4; CRP, C-reactive protein. | | | | | | | | | | | | | |
